# Fine-scale analysis of an assassin bug's behaviour: predatory strategies to bypass the sensory systems of prey

**DOI:** 10.1098/rsos.160573

**Published:** 2016-10-26

**Authors:** Fernando G. Soley

**Affiliations:** 1 Department of Biological Sciences, Macquarie University, Sydney, New South Wales, Australia; 2 Escuela de Biología, Universidad de Costa Rica, San José, Costa Rica

**Keywords:** araneophagy, *Stenolemus giraffa*, spider webs, Reduviidae

## Abstract

Some predators sidestep environments that render them conspicuous to the sensory systems of prey. However, these challenging environments are unavoidable for certain predators. *Stenolemus giraffa* is an assassin bug that feeds on web-building spiders; the web is the environment in which this predator finds its prey, but it also forms part of its preys' sophisticated sensory apparatus, blurring the distinction between environment and sensory systems. *Stenolemus giraffa* needs to break threads in the web that obstruct its path to the spiders, and such vibrations can alert the spiders. Using laser vibrometry, this study demonstrates how *S. giraffa* avoids alerting the spiders during its approach. When breaking threads, *S. giraffa* attenuates the vibrations produced by holding on to the loose ends of the broken thread and causing them to sag prior to release. In addition, *S. giraffa* releases the loose ends of a broken thread one at a time (after several seconds or minutes) and in this way spaces out the production of vibrations in time. Furthermore, *S. giraffa* was found to maximally reduce the amplitude of vibrations when breaking threads that are prone to produce louder vibrations. Finally, *S. giraffa* preferred to break threads in the presence of wind, suggesting that this araneophagic insect exploits environmental noise that temporarily impairs the spiders' ability to detect vibrations. The predatory behaviour of *S. giraffa* seems to be adaptated in intricate manner for bypassing the sophisticated sensory systems of web-building spiders. These findings illustrate how the physical characteristics of the environment, along with the sensory systems of prey can shape the predatory strategies of animals.

## 1. Introduction

Prey are generally well equipped for detecting predators [1,2], which means that predators face the challenge of remaining concealed as they approach or attack. Such challenge proves more or less difficult depending upon the surrounding physical environment and the sensory capabilities of prey [2,3]. These factors have most probably shaped the evolution of predatory strategies in the same way that they have shaped the evolution of animal signals for communication (e.g. [4,5]), albeit commonly in the opposite direction. While signals between communicating animals have evolved to be effectively transmitted in the environment to stimulate the receiver, predators usually strive to minimize the transmission of cues that could alert prey [6,7].

As there are environments that are favourable for signal transmission, there are environments that are unfavourable for launching predatory attacks. For instance, lions prefer to hunt in areas with enough cover to conceal their movements (i.e. areas with higher prey ‘catchability’), rather than in open areas, which despite having greater prey densities, render the lions conspicuous to prey [8]. However, some environments are unavoidable and can be especially challenging for predators. Consider the group of predators that specialize on eating web-building spiders (i.e. araneophagic insects and spiders) [9–11]. A spider's web is not only the environment where such predators find their prey, it is also an intimate part of their prey's main sensory apparatus, through which they detect telltale vibrations from intruder movements [12–14].

Some araneophagic predators avoid alerting their prey by minimizing contact with the web; for instance, jumping spiders that suspend themselves from a dragline to reach the spider at the hub of the web [15,16], or damselflies that use their superb flying ability to glean spiders off webs [11]. Similar to many predators, these araneophagic predators tend to circumvent their prey's highly developed sensory apparatus. Surprisingly, while lacking the advantages conferred by flight or silk, the emesine assassin bugs (Heteroptera: Reduviidae) of the genus *Stenolemus*, specialize on eating web-building spiders [17–19]. Because *Stenolemus* approaches spiders on foot, it needs to accomplish what appears like two mutually exclusive tasks: bypass their sophisticated prey's sensory system at the same time that they approach through it. These assassin bugs occasionally fall prey to the very spiders they pursue, which highlights their risky lifestyle [18,19].

*Stenolemus giraffa* captures the spiders at the resting sites of their webs (i.e. the hubs of orb-weavers, or the top of dome-shaped webs in other spider families), so that it needs to remain concealed as it approaches all the way from the web's periphery to the spider's resting site. However, *S. giraffa* is a large insect (body length of adults around 2 cm, leg span around 5 cm) and routinely needs to break threads that obstruct its path to the spiders [19]. Breaking threads is dangerous, as it requires direct manipulation of the silk, and hence the production of vibrations. Nevertheless, the breaking of threads commonly appears to go unnoticed by the spiders [19,20]. This is especially surprising when *S. giraffa* breaks threads next to the spider [21]. Behavioural observations suggest that *S. giraffa* lowers the amplitude of vibrations produced when breaking threads by holding on to the loose ends of the broken thread and causing them to sag prior to release [19]. This study tests this hypothesis by measuring vibrations directly.

The threads in a web are under different pretensile forces [22,23] and are therefore likely to vary in the amplitude of the vibrations produced when broken. For example, thick threads from the frame of the web have higher pretensile forces than thin radial threads [22]. In addition, threads under higher tension transmit vibrations with lesser attenuation than sagging threads [13,24]. Therefore, the risk of alerting the spiders varies according to the particular characteristics of the threads being broken. It can thus be expected for *S. giraffa* to behave differently when breaking threads that are prone to produce loud vibrations or transmit them more effectively. In addition, the risk of alerting the spiders can be ameliorated under certain conditions. For instance, wind acts as environmental noise that temporarily impairs the spiders' ability to detect vibrations generated by intruder movements [25–27], so that *S. giraffa* could benefit by breaking threads mostly in the presence of wind. For these reasons, this study also evaluates how *S. giraffa* addresses the varying challenges posed by different thread-breaking contexts.

## 2. Material and methods

Juvenile (third–fifth instar) and adult *S. giraffa* (hereafter ‘bugs’) were collected from rock escarpments in close vicinity to El Questro Station, East Kimberley Region, Western Australia (15°53.675′ S, 128°07.986′ E and 16°01.275′ S, 128°01.395′ E), and kept in a controlled environment in Sydney (20–26°C; 60–85% RH, 12 L : 12 D cycle; see the electronic supplementary material, S1 for housing details). To complement the laboratory experiments detailed below, breaking of threads by bugs was also observed under natural conditions at El Questro Station (refer to the electronic supplementary material, S2). Assassin bugs were collected under permits SF007117 and SF007584 from the Department of Environment and Conservation of Western Australia.

### 2.1. Artificial webs and experimental set-up

The use of artificial webs and laser vibrometry was implemented to obtain clean recordings of the vibrations produced by the bugs when breaking individual threads. In nature, the properties of individual threads (e.g. length, diameter, tension, orientation, number of connections to other threads) vary considerably within a single web, so that vibrations recorded in natural webs do not allow for the direct comparisons required in this study. As the noise generated by the spiders themselves also complicates the task of assigning particular vibrations to the specific movements used for breaking threads, artificial webs that were decoupled from the spiders were used to standardize thread condition and to obtain accurate measurements that could be compared between treatments.

**Figure 1.**
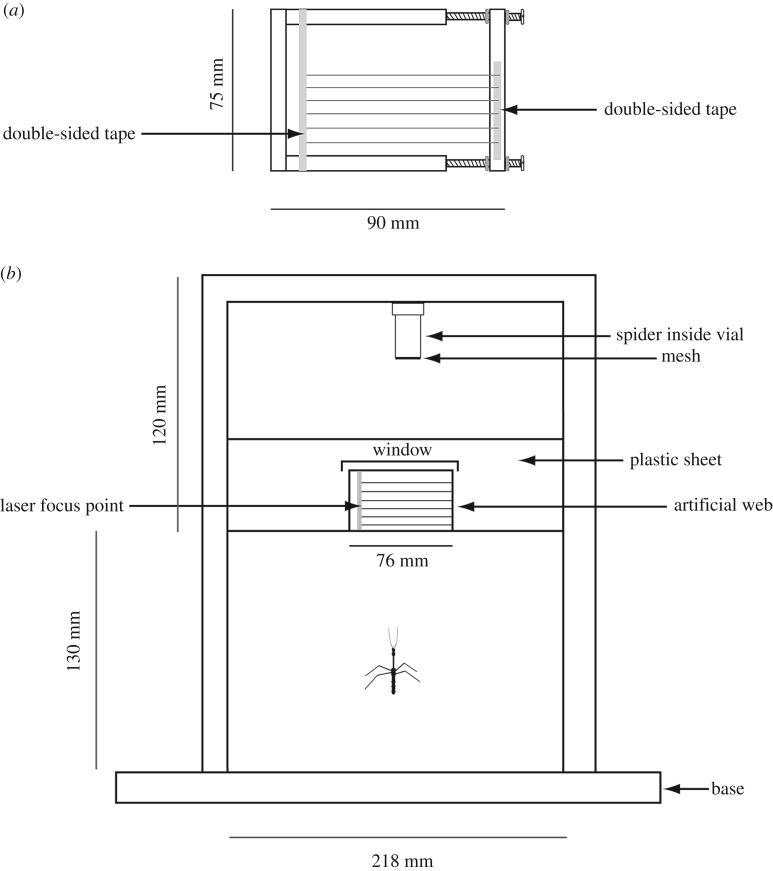
(*a*) Artificial web and (*b*) experimental set-up for measuring the vibrations produced when breaking threads.

The spiders used for building artificial webs were *Pholcus phalangioides* (Pholcidae) collected from the vicinity of Macquarie University in Sydney, Australia; previous observations indicated a very high degree of similarity in the predatory sequence of *S. giraffa* pursuing *P. phalangioides* and its most common prey in nature: pholcid spiders of the genus *Trichocyclus* [19] (FG Soley 2009, personal observation). Artificial webs were constructed using dragline silk collected from spiders that were in ‘good’ body condition (globoid abdomens). One spider was used to build each web, and each web consisted of six lines of silk, separated from each other by around 5 mm (figure 1).

Draglines were extracted in the laboratory (25–28°C, 60–70% RH) by placing a *P. phalangioides* close to the edge of a shelf and nudging it with a brush so that it dropped 145 mm to the shelf underneath. The shelf edges were covered with double-sided adhesive tape to facilitate attachment of the draglines. The dragline left between the two shelves was collected with a wooden frame that had two pieces of double-sided adhesive tape (3 mm width) at the attachment points. One of the pieces of double-sided tape was stuck along the frame's vertical, right border; whereas the other piece was only attached to the horizontal borders of the frame, causing the thread to remain suspended within the frame (figure 1). This procedure was repeated until the artificial web was completed. Draglines stuck to the two pieces of adhesive tape, and also to the frame's vertical border on the left. To ensure that the threads were only suspended from the pieces of adhesive tape, the attachment of each thread to the vertical border on the left was broken by pressing the thread perpendicularly with a ruler, at the section between the piece of tape on the left and the left border of the frame.

To focus the laser vibrometer, a dot of silver reflective paint was applied on the adhesive tape, in between the attachment points of silk lines 3 and 4 (figure 1). By turning screws that held one side of the frame, the attachment points could either be brought further apart or closer together to manipulate web tension (figure 1). Screws were either tightened or loosened four full turns to decrease or increase the tension, respectively. For each trial, the web consisted of draglines from either small (mean = 2.4 mg; range 1–8 mg) or large spiders (mean = 29.8 mg; range 18–40 mg; i.e. thin and thick draglines, respectively), and the tension in these threads was increased, decreased (causing the threads to sag slightly) or left as when collected (i.e. original tension).

The wooden frame containing the web was placed on the top of a rectangular sheet of white corrugated plastic (101 × 218 mm) that was attached to the experimental apparatus and had a window cut open so that the full web could be exposed. The plastic sheet with the web resting on top was placed at an angle of 45° from the bench and measured from the back of the experimental apparatus. The web was oriented horizontally (i.e. threads parallel to the ground), and placed *en route* to a live *P. phalangioides* that was at the top of the set-up and functioned as ‘a lure’ (figure 1); there is some evidence that suggests these bugs use chemical cues to locate the spiders in their webs under natural conditions [19–21]. The spider was inside a semi-clear plastic vial (25 ml) that had the bottom removed and replaced by black fibreglass insect screening. A cotton ball was placed halfway inside the vial after introducing the spider to ensure the spider remained at the bottom and also to provide a substrate from which the spider could hang. Lure spiders were well fed and of intermediate size (weight mean = 12.7 mg; range 3–45 mg). Only one spider was used during each day as a lure, and up to six trials were performed in 1 day.

### 2.2. Recording of behaviour and vibrations

One hour before trials began, each bug was transferred into a 50 ml plastic vial. The inside of the vial was lined with a piece of paper that served as walking substrate. To begin a trial, the piece of paper on which the bug stood was removed from the vial and gently placed at the centre of the wooden base in the experimental apparatus (figure 1). The bugs assumed a resting posture inside the vial, but usually became active within a few seconds after placing them in the experimental apparatus. If the bugs failed to contact the web within 30 min, the trial was aborted. Trials were only considered for analysis if the bug tapped the web with its antennae, a behaviour that is strongly associated with pursuit of spiders [19,20].

A video camera (DCR-TRV22E with supplementary close-up lenses; Sony, Japan) and a laser vibrometer (Polytec PDV100, Germany) were placed 30 cm away from the experimental set-up, each mounted on a different tripod. The laser was set perpendicularly to the set-up, so that the focusing point hit the reflective dot at an angle of 45° from the piece of tape. Video recordings (25 frames s^−1^) were digitized to hard drive in MP4 format through a Digital Rapids DC 1500 A–D board, using STREAM v. 1.5.23 software (Digital Rapids, Canada). Vibrations were recorded at 44.1 kHz per 16 bits, with a low-pass filter set at 22 kHz. The AES (Audio Engineering Society) output of the laser vibrometer was converted to EBU (CO3, Midiman, M-Audio, USA) and synchronized to the audio of video recordings. To keep the laser vibrometer in focus, its analogue output was monitored using headphones connected to a Eurorack UB 802 soundboard (Behringer, Germany).

### 2.3. Vibrations used as a baseline for comparison

The same artificial web design described above was used to establish baseline data of vibrations generated when silk is broken without behavioural amelioration. Two initial approaches were used. During preliminary procedures only, threads were cut with a gentle upward movement of a sharp dissecting blade. However, breaking threads with a hot wire proved more effective, as this method avoided direct contact with the silk (threads snapped before contact). The hot wire consisted of a small piece (7.5 cm) of iron wire (0.6 mm) embedded into a wooden handle (7 cm); the tip of the wire was heated by putting it directly under the flame of a candle until it turned red. Immediately after, the wire's tip was gently brought near a thread of the artificial web. If the wire had been properly heated until it turned red, the thread snapped just before contact (otherwise the thread snapped only when touched). Because contact with the thread was not made, the vibrations recorded at the moment of breaking were attributed to the sudden release of potential energy stored in the thread.

To determine whether distance from the focus point of the laser vibrometer had an effect on the measured amplitudes (i.e. if attenuation along the horizontal axis of the thread was significant), threads were broken with the hot wire at distances ranging from 6 to 61 mm away from the focus point of the laser vibrometer. Breaking distances to the focus point were measured from video images, using ImageJ v. 1.44 software (National Institutes of Health, USA); a 10 mm scale that was drawn on the experimental set-up was used as length reference.

### 2.4. Measurement of vibrations

Vibrations were analysed with Raven Pro 1.4 (Cornell Lab of Ornithology, USA). Preliminary waveform analysis revealed that vibrations produced when breaking or touching threads were in the 70–340 Hz range; therefore, a bandpass filter (0–3000 Hz) was applied to all recordings to reduce high-frequency noise. Peak-to-peak amplitude (hereafter ‘peak amplitude’) was measured directly from the waveform. Peak-to-peak amplitudes are biologically relevant because naturally occurring vibrations need to reach threshold levels in order to stimulate spider receptors [13,14]. Measurements of peak-to-peak amplitude are not affected by background noise levels, as long as the vibration of interest is louder than the noise.

The bugs sometimes obstructed the laser focus point with their bodies, or broke threads while stepping on other threads or on the tape that the laser was focused on; these, and vibrations that occurred when the laser was out of focus, were excluded from analysis. Amplification was occasionally necessary to accurately locate the moment the thread broke, but the measurement was always taken from the unamplified version.

### 2.5. Breaking threads in the presence of background noise

To determine whether the bugs were more likely to break threads in the presence of background noise from wind, a stand fan was placed 1.5 m away from the experimental set-up described above and at the same height. The fan rotated 90° to the sides at 0.1 cycles s^−1^ and it faced the experimental set-up at 45° when it was at half this trajectory. The wind speed measured at the artificial web when the fan was facing it without rotating (i.e. the maximum wind speed experienced by the web during the fan's trajectory) was 0.1 m s^−1^, which is at the lower end of the wind speeds measured during spider pursuits by *S. giraffa* in the field (mean = 0.3 m s^−1^; range = 0.1–1.8 m s^−1^; *n* = 35) [19].

For this experiment, the bugs were assigned randomly to one of the two treatments (i.e. ‘wind’, ‘no wind’) and were tested the following day on the other treatment. The bugs were placed at the base of the set-up as usual, but the fan was turned on when they started climbing up towards the artificial web. In the ‘no-wind’ treatment, the fan was turned off just before the bug contacted the web with its antennae; in the ‘wind’ treatment, the fan was left on until the trial ended. Only small spiders were used to build the artificial webs in this experiment, and thread tension was not manipulated.

### 2.6. Statistical analysis

Peak amplitude data were analysed using generalized linear mixed-effects models. First, using likelihood ratio tests, the significance of the following fixed effects and interactions was tested for in the baseline only: silk origin (i.e. small or large spiders), thread tension, thread position within the web, number of threads left in the web at the moment of breaking, horizontal distance (to the laser's focusing point) of the breaking point; and the interactions: silk origin × thread tension and horizontal distance of the breaking point × thread tension. Bug ID and spider ID were included as random effects in all models. Non-significant fixed effects and interactions were removed from the final model for subsequent comparisons with the peak amplitudes produced by the bugs.

Generalized linear mixed-effects models were also used to compare the duration of different components of thread-breaking behaviour of the bugs according to the thread's size and tension and if there was wind present. Logistic regressions were used to determine whether the bugs were more likely to break a thread in the web, depending on silk origin and thread tension. Logistic regressions were carried out using the ‘lmer’ function of the package ‘lme4’ in the open source R software. Generalized linear mixed-effects models and post hoc comparisons were carried out using the functions ‘glmer’, ‘glmmPQL’ and ‘glht’ of the packages ‘lme4’, ‘MASS’, and ‘multcomp’, respectively, in the open source R software.

## 3. Results

### 3.1. Vibrations used as a baseline for comparison

Peak amplitude of vibrations produced in the baseline did not vary significantly with horizontal distance from the laser focus point or the number of threads left in the web at the moment of breaking (range 1–6; table 1); therefore, these factors were excluded from the model in subsequent comparisons. The thread's position within the web (i.e. if it was the thread at the base of the web, the top of the web or one of the four intermediate positions) had a significant effect in the model (table 1). There was a marginally significant interaction between spider size and thread tension (table 1); this interaction was kept while testing for fixed effects. There was no significant interaction between thread tension and the horizontal distance at which the thread was broken from the laser focus point (i.e. horizontal attenuation of the vibration did not differ significantly between threads under different tension; table 1).

**Table 1.** Significance of fixed effects and interactions included in the general linear models used to explain variation in the peak amplitude of vibrations produced when (*a*) breaking threads of large and small spiders under different tension in the baseline; (*b*) breaking threads of large spiders under different tension in two treatments (bugs or baseline); (*c*) breaking threads of small spiders under different tension in two treatments (bugs or baseline). In (*a*), the interaction of spider size by tension was marginally significant; therefore, it was kept in the models when testing for significance of fixed effects. Statistical significance in probability tests is indicated by asterisks [28].

	*p*-value
(*a*) baseline	
interaction of spider size by tension	0.056
interaction of distance by tension	0.393
thread's position	0.013*
number of threads left	0.204
horizontal distance to laser focus point	0.382
(*b*) large spiders (bugs versus baseline)	
interaction of treatment by tension	0.143
treatment	<0.001*
tension	0.033*
(*c*) small spiders (bugs versus baseline)	
interaction of treatment by tension	0.029*

In webs from large spiders (but not in webs from small spiders), breaking of sagging threads produced vibrations of lower peak amplitudes than breaking of threads of original and increased tensions (table 2). Owing to this interaction between spider size and thread tension, comparison of baseline vibrations with vibrations produced by the bugs was carried out separately for webs from large and small spiders.

**Table 2.** Post hoc Tukey comparisons of mean peak amplitudes produced when breaking threads under different conditions in (*a*) the baseline, (*b*) when breaking threads of large spiders and under different tension in two treatments (bugs or baseline) and (*c*) when breaking threads of small spiders and under different tension in two treatments (bugs or baseline). The sign of the estimate refers to the factor on the left for each pairwise comparison. Statistical significance in probability tests is indicated by asterisks [28].

	estimate	s.e.	*z*-value	*p_r_* (>|*z*|)
(*a*) baseline				
*threads from small versus large spiders*				
all under increased tension	−2831.6	541.96	−5.225	<0.001*
all under original tension	−2780.9	474.4	−5.862	<0.001*
all sagging	−1374.9	472.9	−2.907	0.042*
*threads from large spiders*				
sagging versus original tension	−1496.9	477.2	−3.137	0.021*
increased tension versus original tension	306.6	496.9	0.617	0.990
increased tension versus sagging	1803.5	500.7	3.602	0.004*
*threads from small spiders*				
sagging versus original tension	−90.9	468.4	−0.194	0.999
increased tension versus original tension	255.9	521.5	0.491	0.997
increased tension versus sagging	346.8	516.9	0.671	0.985
(*b*) treads from large spiders: bugs versus baseline				
bugs versus baseline	−1635.9	334.9	−4.885	<0.001*
(*c*) threads from small spiders: bugs versus baseline				
increased tension	−1342.4	455.5	−2.947	0.035*
original tension	−1055.8	413.9	−2.551	0.104
sagging	−313.8	412.0	−0.762	0.972

### 3.2. Breaking of threads by the bugs

The bugs treated the artificial webs in apparently the same manner as they treat natural webs [19]. Usually, the bugs first waved their antennae in the direction of the artificial web and the spider, and then walked slowly towards them. Once in front of the web, the bugs leaned over to ‘tap’ the silk threads with their antennae, while keeping their body and legs away from the web (electronic supplementary material, video). When tapping, these bugs bring their antennae very close to the web, but it is not clear if they contact the silk [19]. After this first stage, the bugs either decamped from the site, or placed one or both of their foretarsi on the web (i.e. ‘grab’ the thread). Placement of foretarsi on threads was done softly and quietly (i.e. no vibrations could be detected; see below). After placing one or both foretarsi on the web, the bugs decamped from the site, climbed up the web (by placing middle and hindlegs on the web) or broke one or more threads from the web.

The bugs used two tactics to break threads with their forelegs, ‘reckless’ (7% of breakages; *n* = 152) and ‘cautious’ (93% of breakages). When using the reckless tactic, the bugs grasped the thread with their foretarsi (sometimes with foretibiae) and pulled it forcefully and perpendicular to the thread's longitudinal axis by flexing all their legs. Only threads from small spiders were successfully broken using the reckless tactic (three out of four pulling instances). The bugs also used the reckless tactic on threads from large spiders (six pulling instances), although none of these threads broke (although the tension appeared to have been lessened in some of these threads). More often, the bugs used the cautious tactic. This tactic involved positioning the foretarsi closer together than in the reckless tactic (so that the foretarsi touched or almost touched each other), and then pulling them away from each other along the longitudinal axis of the thread (as opposed to perpendicular pulling in the reckless tactic), until it broke (electronic supplementary material, video). On rare occasions, the bugs needed to repeat these movements in order to break the thread. When the thread broke, the bugs did not let it snap back into the web, but instead held both loose ends (one with each foreleg), usually for several seconds and occasionally for minutes, before releasing them (electronic supplementary material, S1—figure S1). The bugs usually extended their forelegs towards the attachment points of the thread (one foreleg at a time), causing the loose ends to sag before releasing them. There was no evidence of the bugs releasing first the shorter or the longer loose ends of the threads (binomial test, *p* = 0.42, *n* = 24).

When the bugs broke threads, they usually continued their path towards the spider inside the vial (86%, *n* = 62), while minimizing contact with the remaining threads (electronic supplementary material, video). Otherwise, the bugs began to rest or decamped from the site. Once on the other side of the web, the bugs often (53%, *n* = 53) arrived to tap the mesh of the vial holding the spider, or otherwise continued walking around the experimental apparatus.

### 3.3. Attenuation of vibrations by the bugs

**Figure 2.**
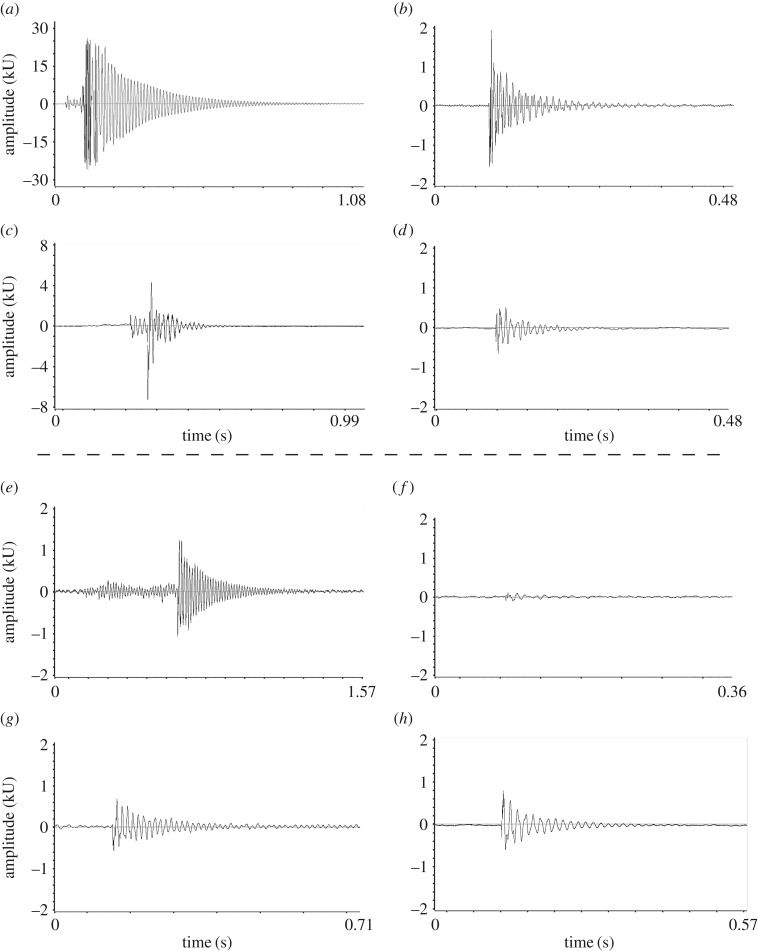
Representative waveforms of the vibrations produced when breaking threads by (*a*) cutting with a surgical blade, (*b*) nearing a hot wire, (*c*) *Stenolemus giraffa* using the reckless tactic (in this case the thread did not break), (*d*) *S. giraffa* using the cautious tactic, (*e*) *S. giraffa* using the reckless tactic, (*f*) *S. giraffa* using the cautious tactic, (*g*) *S. giraffa* using the cautious tactic, but failing to hold on to the loose ends of the broken thread. (*h*) A vibration produced by a thread suddenly becoming detached from one of its attachment points. Threads above the dotted line (*a*–*d*) are from large spiders and threads below it (*e*–*h*) are from small spiders. All threads were taut (i.e. original or increased tension), except the thread in ‘*h*’, which was sagging. Note scale differences in (*a*) and (*c*).

**Figure 3.**
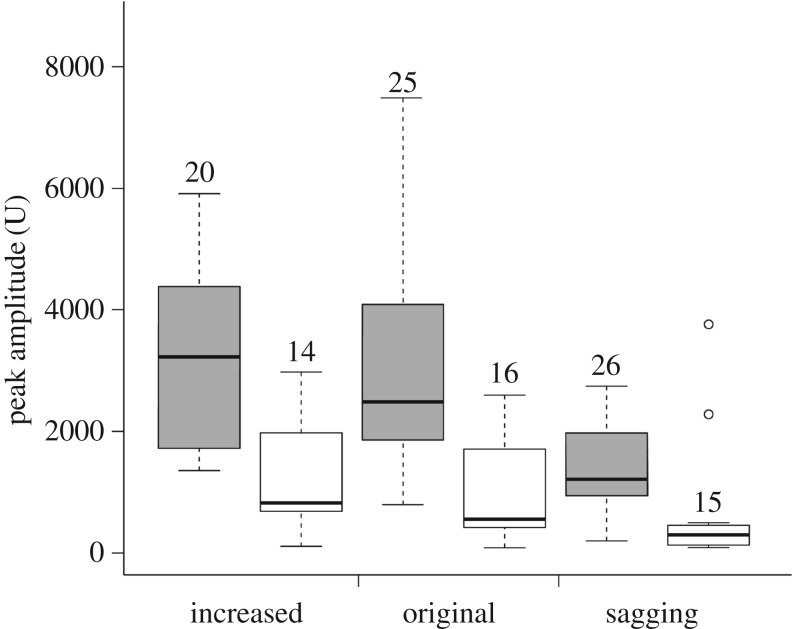
Peak amplitude of the vibrations produced when breaking threads from large spiders that were under different tension. Cautious breaking by the bugs is shown in white; breaking with the hot wire is shown in grey. Boxes denote median, first and third quartiles; whiskers denote the range. Numbers above boxes indicate sample sizes, which consist of repeated measures of 7–9 bugs or 5–6 webs. Open circles denote two instances in which the bugs made a sagging thread become taut when grabbing it; the uppermost circle also refers to a case in which the bug failed to hold on to the loose ends of the broken thread.

**Figure 4.**
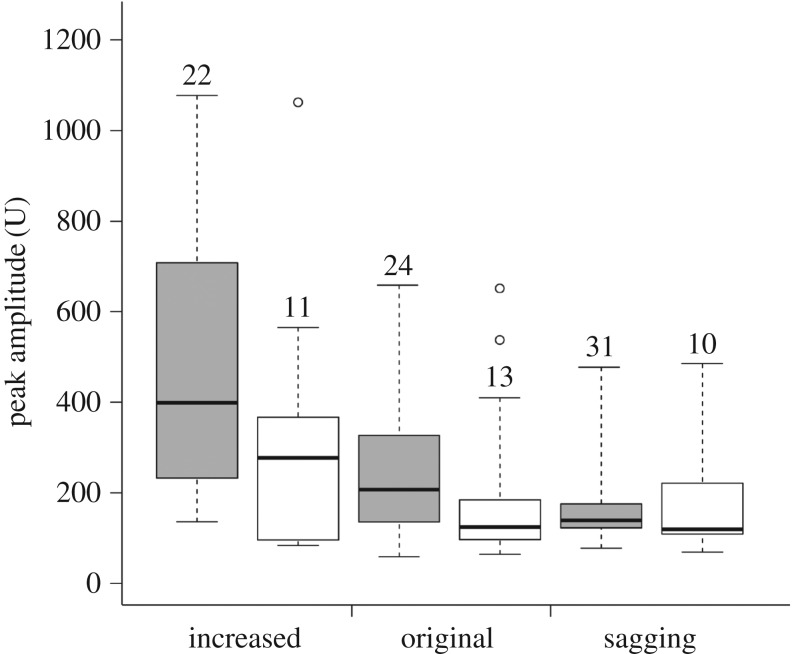
Peak amplitude of the vibrations produced when breaking threads from small spiders that were under different tension. Cautious breaking by the bugs is shown in white; breaking with the hot wire is shown in grey. Boxes denote median, first and third quartiles; whiskers denote the range. Numbers above boxes indicate sample sizes, which consist of repeated measures of 7–10 bugs or 4–6 webs. Open circles denote instances in which the bugs failed to hold on to the loose ends of broken threads.

There were only a few instances in which the bugs produced loud vibrations when breaking threads; these were when the bugs used the reckless tactic or when they failed to hold on to the loose ends of broken threads (these were considered as outliers and removed from further analyses; see below). When the bugs broke threads with the reckless tactic, the vibrations produced were much louder than those produced in the baseline of breaking threads with a hot wire (figure 2). On four occasions, the bugs broke a thread using the cautious tactic, but failed to hold on to one or both loose ends of the broken thread; in such cases, the peak amplitude of the vibrations produced were similar to those produced in the baseline (outliers in figures 3 and 4; also cf. figure 2*g*,*h*).

When breaking threads from large spiders using the cautious tactic, the bugs produced vibrations of significantly lower peak amplitudes than the baseline, regardless of thread tension (tables 1 and 2; figure 3). When breaking threads from small spiders using the cautious tactic, the bugs produced vibrations of significantly lower peak amplitudes than the baseline if the thread was under increased tension (a similar tendency was observed for threads under original tension, but this was not statistically significant; tables 1 and 2; figure 4). When breaking threads from small spiders that were sagging, the peak amplitude of the vibrations produced by the bugs did not differ from the baseline (tables 1 and 2; figure 4).

The vibrations produced when the bugs released the left loose end of broken threads were very soft and only occasionally distinguishable from background noise (17% of recordings, *n* = 87). When detected, these vibrations were very similar for threads of large and small spiders (electronic supplementary material, S1—figure S2). The right loose end was attached to the wooden frame and vibrations on this end could not be measured.

Tapping the web with antennae, placing foretarsi on threads and removing foretarsi off threads could not be detected in any of the recordings, even in the cleanest ones with minor noise levels and after amplifying up to 54 dB. Other bug movements (e.g. hitting a thread with the tibiae of middle and hindlegs, stepping onto a thread with middle and hindlegs) were only occasionally detected in the recordings; when detected, these vibrations were of very low amplitudes (mean peak amplitude: 101.4 kU, s.d. = 46.4; *n* = 13; see the electronic supplementary material, video).

### 3.4. Breaking threads in the presence of wind

**Figure 5.**
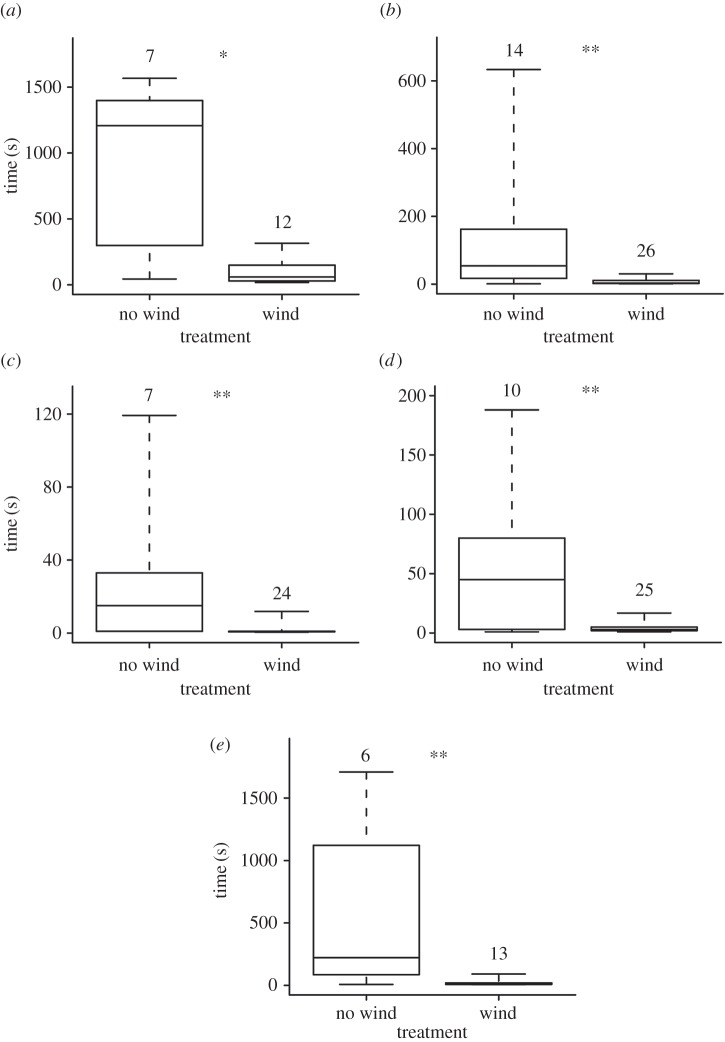
Time elapsing between different components of the bugs' thread-breaking behaviour in the absence or the presence of wind: (*a*) time to break first thread after tapping; (*b*) time to break first thread after grabbing; (*c*) time to release the first loose end of a broken thread; (*d*) time to release both loose ends of a broken thread; (*e*) time elapsed between consecutive breaking of threads. Boxes denote median, first and third quartiles; whiskers denote the range. Numbers above boxes indicate sample sizes, which consist of repeated measures of 4–11 bugs. *p < 0.05; **p < 0.01.

The bugs were more prone to break threads if there was wind present: nine bugs broke threads only when wind was present, four bugs broke threads in both treatments, two bugs did not break threads in either treatment, and no bug broke threads in the no-wind treatment only (McNemar's test = 7.11, d.f. = 1, *p* < 0.01, *n* = 15). The duration of the measured components of thread-breaking behaviour (i.e. tapping, grabbing and breaking threads) were all affected by the presence of wind (figure 5). The time elapsing between first contact of silk with antennae (i.e. tapping) and breaking of the first thread, and the time elapsing between first contact of silk with foretarsi (i.e. grabbing) and breaking of the first thread, were significantly shorter in the presence of wind. In addition, the bugs took less time to break consecutive threads and released the loose ends of broken threads sooner in the presence of wind.

## 4. Discussion

### 4.1. Attenuation of vibrations by *Stenolemus giraffa*

*Stenolemus giraffa* can actively reduce the amplitude of the vibrations produced when breaking threads in webs. The bugs achieved this by using a cautious breaking tactic that involved holding on to the loose ends of broken threads and causing them to sag prior to release. In the few cases in which the bugs did not hold on to the loose ends of the broken thread, the vibrations produced were louder and similar in amplitude to the vibrations in the baseline, indicating the importance of this behaviour to prevent a sudden release of energy when the thread breaks. Unlike in the baseline, however, breaking of threads by the bugs necessitated placement of extra weight on the thread (i.e. grabbing the thread with foretarsi), which loads the thread and thereby increases the force which it is under [22]. Therefore, it could be expected for vibrations produced by the bugs to be naturally louder than the ones produced in the baseline. Yet, the bugs produced vibrations of smaller or otherwise similar peak amplitudes than the baseline.

In the case of threads from large spiders, the bugs produced vibrations of significantly lower peak amplitudes than the baseline, regardless of tension. The same was true for threads from small spiders that were under increased tension (and a non-significant tendency in the same direction was also observed for threads under original tension). Sagging threads from small spiders were probably too thin to support the weight placed by the forelegs of the bugs, so that a significant increase in the tension of these threads might have caused the vibrations to be similar in amplitude to the ones produced in the baseline. Even for threads from large spiders, it was evident in at least two cases that the sagging threads became taut as soon as the bugs grabbed them (see outlier values in figure 3).

In the baseline, threads from large spiders produced louder vibrations at the moment of breaking than did threads from small spiders. This suggests that the bugs are at greater risk when breaking threads from large spiders. Given this higher risk, it was expected that the bugs behaved rather carefully when breaking threads from large spiders (e.g. took more time before deciding to break a thread, or took more time to release the loose ends of a broken thread), and that they more often avoided breaking such threads. Because taut threads also produced louder vibrations (at least for threads of large spiders), it was also expected that the bugs behaved more carefully when breaking taut threads (and that they more often avoided breaking taut threads). However, the bugs' decision of breaking a thread in the artificial web was not affected by the tension of the threads, or if these came from large or small spiders. In addition, the duration of the different components of thread-breaking behaviour were not influenced by the thread's tension or if it came from a large or small spider (electronic supplementary material, S1—table S1). However, the attenuation of vibrations by the bugs was maximal for threads of large spiders and for taut threads (compare the reduction of amplitudes in figures 3 and 4). Perhaps this differential reduction in amplitude is achieved by distinctive joint movement or muscular activity, which are variables that were not assessed in this study. Regardless, it is important to note that the amplitude reduction was maximal for the threads that tend to produce louder vibrations. Still, it appears that there are limits to the reduction in amplitude that the bugs can achieve, because the vibrations that they produced when breaking these threads were still louder when compared with the vibrations from other threads.

### 4.2. The stealthy tactic of *Stenolemus giraffa*

The quiet thread-breaking behaviour of *S. giraffa* goes in hand with the overall stealthy tactic of these bugs. First, *S. giraffa* moves slowly and cautiously in the vicinity of webs, minimizing contact with the silk, and therefore severely curtailing the possibility of detection by the spiders [19,21]. The caution of these movements is illustrated here by the fact that no vibrations could be detected for various types of bug movements (i.e. tapping the web with antennae, grabbing and releasing threads with foretarsi). Similarly, vibrations of other bug movements (e.g. hitting threads with middle and hindlegs) only produced small-amplitude vibrations that were difficult to detect without considerable amplification (see the electronic supplementary material, video). Also, by interspersing periods of quiescence in between the different components of thread-breaking behaviour that tended to produce discernable vibrations (i.e. breaking threads, and releasing the loose ends of broken threads), the bugs appear to reduce the likelihood of being detected by the spiders. In a previous experiment, it was shown that when the spiders detected disturbances in the web caused by *S. giraffa*, they commonly ignored these if the bugs became quiescent immediately after the spiders' initial response [20].

Second, the stealthy tactic is also evidenced in that *S. giraffa* minimizes contact with the web by approaching spiders from adjacent, non-web surfaces [21]. However, when approaching through non-web surfaces, *S. giraffa* needs to break more threads (e.g. mooring lines attaching the web to the adjacent surface). An alternative more direct route through which there are no blocking threads is to approach across the web's capture area, but such a route entails a greater risk of detection and death [20,21]. In addition, *S. giraffa* that approach through the ‘safe’ route (i.e. non-web surfaces) usually end up just behind or above the spider, but with the web in between, so that they further need to break threads in close proximity to the spiders [21]. The cautious thread-breaking behaviour of *S. giraffa* enables it to approach spiders across safer routes, even if this entails more breaking of threads and doing so in the immediate vicinity of spiders.

### 4.3. The reckless tactic for breaking threads

The bugs can also break threads using the reckless tactic. The reckless tactic produced very high amplitude vibrations, and in observations in nature the spiders sometimes reacted aggressively to these vibrations (see the electronic supplementary material, S2). It remains unclear why the bugs occasionally use this tactic rather than using the cautious tactic in all circumstances. Spiders often ignore large-scale disturbances in their webs, such as those caused by wind or falling debris [29], and one could regard reckless breaking of threads as a self-generated ‘smokescreen’ tactic [26] used by the bugs to conceal themselves from the spiders. However, the bugs never advanced quickly across the web after using the reckless tactic, a behaviour that would be expected if this tactic constituted a self-generated smokescreen. Reckless breaking of threads could nevertheless be advantageous in the sense that it can sever several threads at once, hindering further transmission of vibrations in a specific area of the web and facilitating the concealment of subsequent movements.

### 4.4. The use of wind as camouflage

The bugs were more prone to break threads in the presence of wind, and all measured components of thread-breaking behaviour were faster when there was wind present. This suggests that the bugs are taking advantage of wind to mask their behaviour, because wind can temporarily impair the spiders' ability to detect web-borne vibrations [26,27]. Estimates of spider responses elicited by thread-breaking behaviour range from 14% in this study (see the electronic supplementary material, S2) to 56% [20]; the latter should be regarded as an overestimate as it considered ‘thread-breaking sequences’ that entail breaking of single threads or breaking of several consecutive threads. These estimates indicate that despite the efforts of *S. giraffa* to remain undetected when breaking threads, this behaviour can be occasionally detected by the sophisticated, vibratory system of web-building spiders. However, *S. giraffa* appears to ameliorate this risk even further by taking advantage of wind that temporarily impairs this sensory system.

Another araneophagic assassin bug, *S. bituberus*, approaches web-building spiders more quickly in the presence of wind and has a higher capture success when it hunts in windy conditions [25]. Similarly, some species of araneophagic jumping spiders (Salticidae) in the genus *Portia* use wind-derived noise as an opportunistic smokescreen to approach spiders in webs [26]. In the absence of wind, these spiders may also generate their own smokescreen by shaking the web in a manner that simulates gusts of wind or other large-scale disturbance of the web [30,31]. The fact that distantly related araneophagic arthropods have evolved the ability to exploit (and even create) environmental noise when approaching spiders in webs clearly illustrates the underlying challenge imposed by the need to approach prey across a physical environment (i.e. the web) that forms part of their prey's sophisticated sensory system.

## 5. Conclusion

The hunting sequence that predators generally follow can be broken down into different stages, typically detection, recognition, approach, attack and capture [3,32]. Predators can fail at any of these stages, and prey have evolved defences that interfere with the predator's strategy at different stages [31,33]. When considering the sensory systems of both predator and prey and the characteristics of the physical environment in which predatory interactions take place, it is evident that different stages represent unequal challenges for different predators. These challenges have most likely been important in shaping the evolution of predatory strategies. In the case of *S. giraffa,* the problems associated with forewarning the prey are drastically amplified, and the result has been the evolution of predatory strategies that rely continuously on stealth.

## Supplementary Material

Electronic Supplementary Material 1–Supporting methods, tables and figures

## Data accessibility

Data are available at the Dryad Digital Repository http://dx.doi.org/10.5061/dryad.vg06c [34].
