# Supplementary material for: Fine-scale analysis of an assassin bug's behaviour: predatory strategies to bypass the sensory systems of prey
Source: R Soc Open Sci. 2016 Oct 26;3(10):160573. doi: 10.1098/rsos.160573 (PMC5099001; doi:10.1098/rsos.160573)
Supplement: Electronic Supplementary Material 1–Supporting methods, tables and figures [file rsos160573supp1.docx]

**Electronic Supplementary Material 1–Supporting methods, tables and figures**

**Material and methods**

*Housing conditions for the laboratory experiments*

In Sydney, the bugs were housed in 1-litre, cylindrical plastic containers (diameter 115 mm, height 110 mm) that were lined with white paper, serving as a walking substrate. As food, each bug was provided one well-fed (globoid abdomen) *Pholcus phalangioides* (Pholcidae) each week. The spiders provided were of approximately the same size (leg span) as the bug. However, during the experimental periods (June, July & November 2010; January & February 2011) bugs were fed only small spiders so that they were more likely to be hungry and to exhibit predatory behaviour [1,2].

**Results**

*Bugs breaking threads of different size and tension in artificial webs*

There was no evidence of the bugs being more prone to break threads from large or small spiders, either after tapping or grabbing the web (table S1). Also, there was no evidence of the bugs being more prone to break threads under a particular tension, either after tapping or grabbing the web (table S1).

The time elapsing between first contact of silk with antennae (i.e. tapping) and breaking of first thread by the bugs was similar in webs from small and large spiders, and threads under different tension (table S1; figure S1). Also, the time elapsing between first contact of silk with foretarsi (i.e. grabbing) and breaking of threads by the bugs was similar in webs from small and large spiders, and threads under different tension (table S1; figure S1). There was no evidence of the bugs releasing the loose ends of broken threads faster or slower, according to the thread's tension or if it came from a small or large spider (table S1; figure S1). The time elapsed between consecutive breaking of threads was also similar for threads from small and large spiders, and threads under different tension (table S1; figure S1).

**ESM1 Table S1**. Significance of fixed effects and interactions included in a) the logistic regression analysis used to explain the likelihood of the bugs breaking a thread in the web, depending on the thread´s tension and whether it came from a small or large spider; and b) the general linear models used to explain variation in the latency between different components of thread-breaking behaviour of the bugs, depending on the thread´s tension and whether it came from small or large spiders. *N* refers to sample sizes (number of trials); the total number of bugs used in these trials was 49.

|  | *p-value* |
| --- | --- |
| a) Logistic regression analyses |  |
| Likelihood of breaking thread(s) after tapping web; *N* = 143 |  |
| interaction of spider size by tension | 0.943 |
| spider size | 0.567 |
| thread tension | 0.282 |
| Likelihood of breaking thread(s) after grabbing web; *N* = 83 |  |
| interaction of spider size by tension | 0.622 |
| spider size | 0.112 |
| thread tension | 0.445 |
| b) General linear models |  |
| Latency to break first thread after first contact of silk with antennae |  |
| interaction of spider size by thread tension | 0.963 |
| spider size | 0.246 |
| thread tension | 0.677 |
| Latency to break first thread after first contact of silk with foretarsi |  |
| interaction of spider size by thread tension | 0.800 |
| spider size | 0.098 |
| thread tension | 0.348 |
| Latency to release first loose end of a broken thread |  |
| interaction of spider size by thread tension | 0.457 |
| spider size | 0.394 |
| thread tension | 0.861 |
| Latency to release both loose ends of a broken thread |  |
| interaction of spider size by thread tension | 0.234 |
| spider size | 0.289 |
| thread tension | 0.884 |
| Latency to break next thread |  |
| interaction of spider size by thread tension | 0.658 |
| spider size | 0.838 |
| thread tension | 0.632 |

**
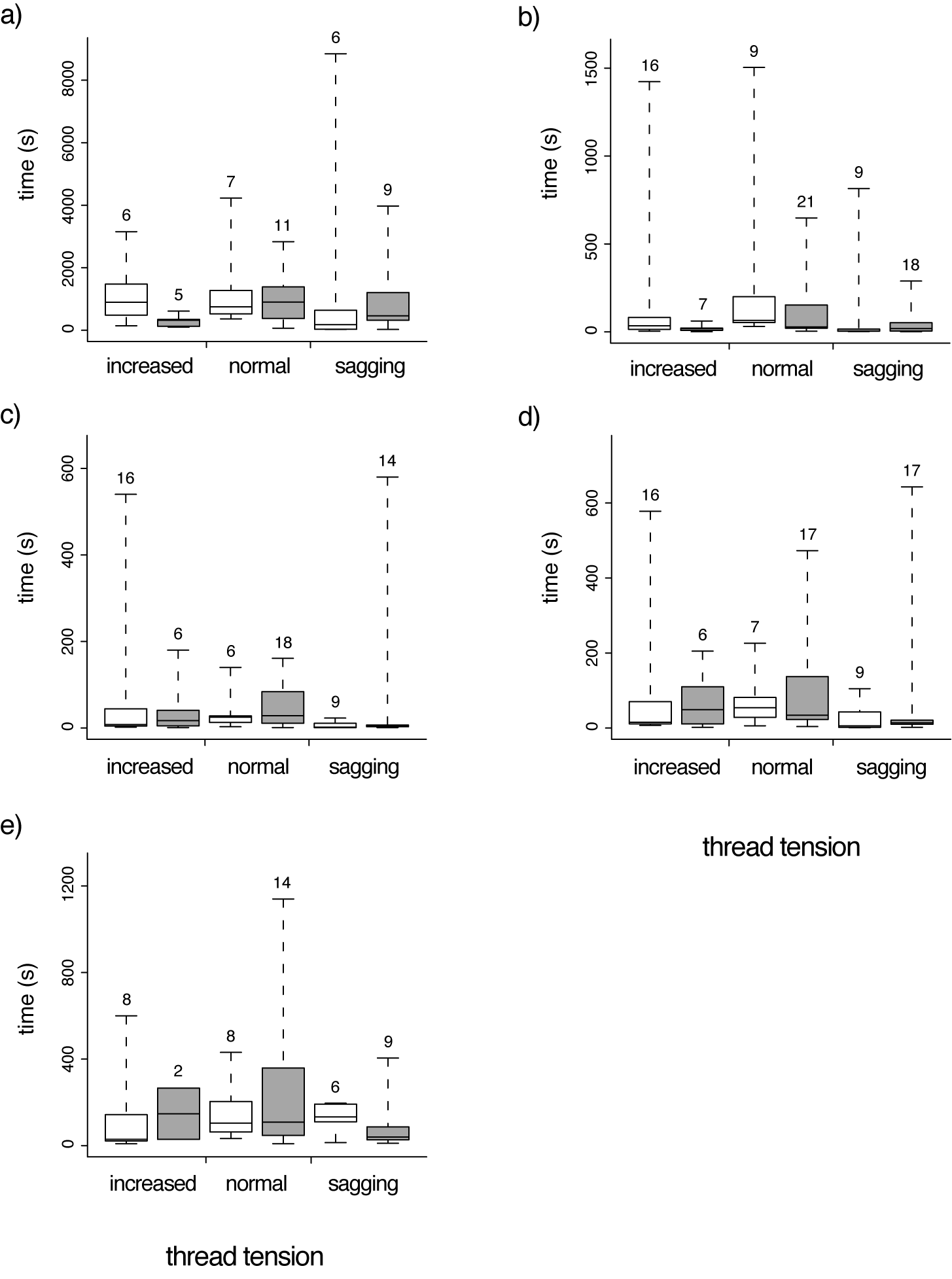
**

**ESM1 Figure S1.** Time elapsing between different components of the bugs' thread-breaking behaviour in webs from large (white boxes) and small (gray boxes) spiders: a) time to break first thread after tapping; b) time to break first thread after grabbing; c) time to release the first loose end of a broken thread; d) time to release both loose ends of a broken thread; e) time elapsed between consecutive breaking of threads. Boxes denote median, first and third quartiles; whiskers denote the range. Numbers above boxes indicate sample sizes, which consist of repeated measures from 2-12 bugs.

**
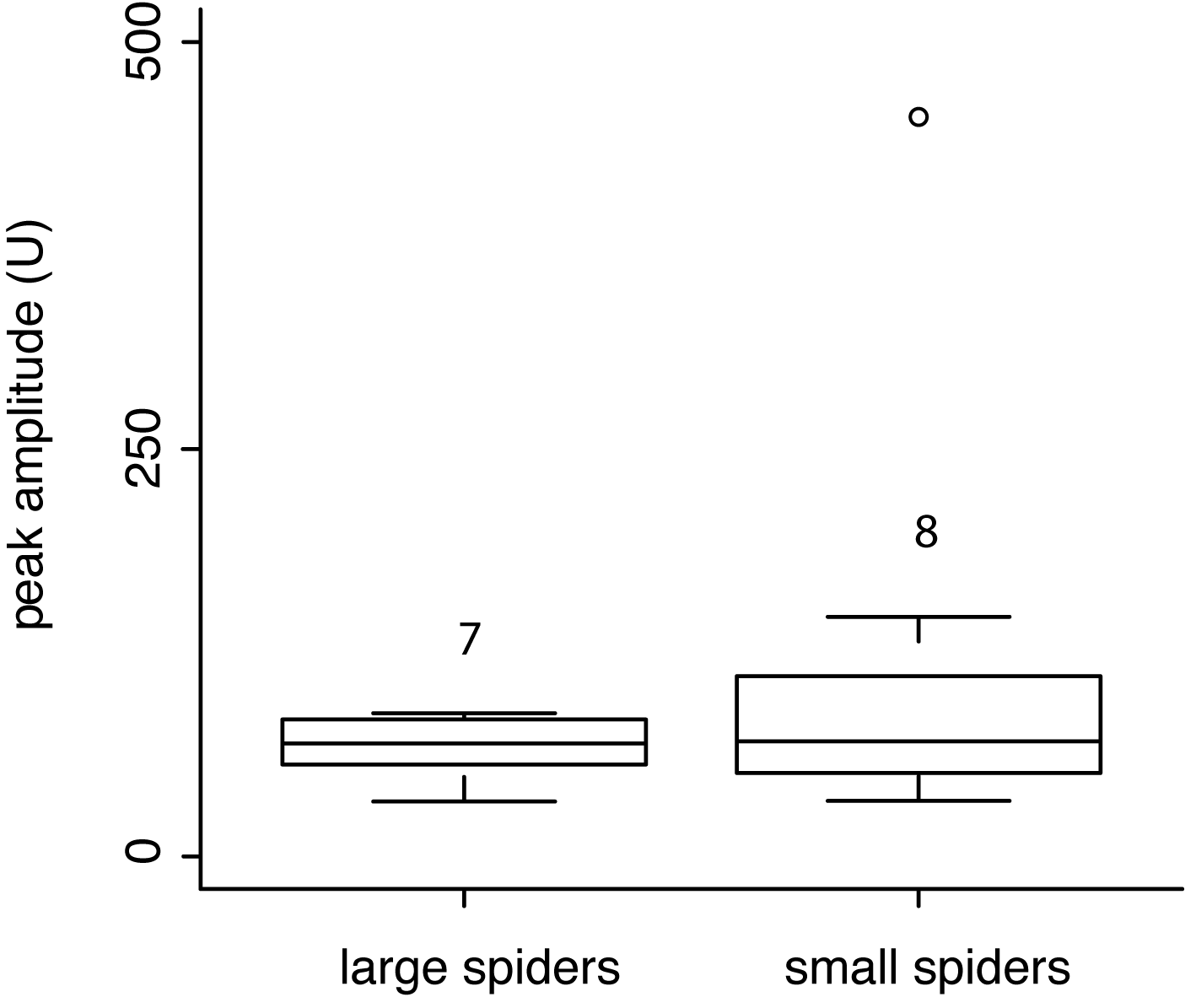
**

**ESM1 Figure S2.** Peak amplitude of the few vibrations that were detected above background noise when the bugs released the left loose end of broken threads, from large and small spiders. Boxes denote median, first and third quartiles; whiskers denote the range; the open circle denotes an outlier value. Numbers above boxes indicate sample sizes.

**References**

1. Soley, F. G., Jackson, R. R., & Taylor, P.W. (2011). Biology of *Stenolemus giraffa* (Hemiptera: Reduviidae), a web invading, araneophagic assassin bug from Australia. *New Zealand Journal of Zoology,* 38, 297-316.
2. Wignall, A. E., & Taylor, P. W. (2009). Alternative predatory tactics of an araneophagic assassin bug (*Stenolemus bituberus*). *Acta Ethologica,* 12, 23-27.

**Electronic Supplementary Material 2–Additional observations in nature**

**Material and methods**

Besides the laboratory experiments, breaking of threads by *S. giraffa* was also observed during staged interactions with three spider species in an open shed at El Questro Station, during August–October 2009 and July–August 2010. The spiders used for interactions were all part of *S. giraffa*'s natural diet [1]: *Trichocyclus arawari* (Pholcidae), *Parasteatoda* sp. (Theridiidae), and *Argiope katherina* (Araneidae). *Trichocyclus* *arawari* and *Parasteatoda* sp. build dome-shaped webs that are suspended by several mooring lines*. Argiope katherina* builds orb webs of vertical orientation*.* The sizes of the spiders used for interactions matched the sizes of spiders that *S. giraffa* pursued in the field [1]. The *A. katherina* used were small and medium-sized juveniles, so that the bug to spider size ratio (body length) ranged from 5:1 to 3:1. The *T. arawari* used were either large juveniles or adults, maintaining a bug to spider size ratio of 2:1 to 1:1 (leg span rather than body length was used to determine bug to spider ratio because pholcids have very small bodies and very long legs).

Interactions were also observed under natural conditions in the surrounding rock escarpments. For a detailed description of the sites and protocols for staging interactions see previous studies [1-3]. The bugs that were used for observing predatory interactions in the field were different individuals from the ones used for the laboratory experiments.

**Results**

During staged and natural predatory interactions, the bugs used the reckless tactic for breaking threads in only 10 occasions out of 150 interactions, and all occurred at the web's periphery; the spiders responded to these in only three occasions: once by leaving the web, once by orienting to the bug, and once by 'bouncing' aggressively [see 3 for a description of 'bouncing']. The bugs never advanced quickly after using the reckless tactic. For the remaining 140 interactions, the bugs broke threads using the cautious tactic. On nine occasions, the bugs were observed to repeat the sequence of movements of the cautious tactic several times (2 to 7) to break a thread that was apparently too thick; six of these occurred when breaking mooring threads from the webs of all spider species. The other three instances occurred in webs of *A. katherina* (once when breaking a frame thread, once when breaking a radial thread, and once when breaking a spiral thread). On two occasions, the bugs were observed to break a thread with the cautious tactic and then fail to hold on to the loose ends, which snapped towards their attachment points.

Observations of predatory interactions suggest that cautious thread-breaking behaviour goes commonly unnoticed by the spiders. Clearly identified instances in which the spider's response (or lack of it) could be associated with the bugs' behaviour, suggest that spiders detected the bugs breaking a thread in their webs in 14% of the occasions (data pulled for all spiders; N = 162 threads broken by 118 bugs; so that the estimate considers repeated measures (2 to 5) for 44 bugs). This estimate is based on instances in which the bug could be clearly observed breaking a thread, and that the spider was at its resting site and had not responded previously with aggressive behaviour [3]. In this way, the spider's behaviour could be associated with more certainty to the bug's behaviour (instead of being related to past behaviour from the bug or the spider itself). This estimate is rough because it considers breaking of different types of threads (e.g. mooring threads, frame threads, threads from the capture area of the web), although threads vary in their likelihood of alerting the spiders (due to differences in their pretensile forces [4]. Similarly, this estimate does not consider the distance from the spider at which the thread was broken, although breaking is more likely to be detected when done in close proximity to the spiders [3].

**References**

1. Soley, F. G., Jackson, R. R., & Taylor, P.W. (2011). Biology of *Stenolemus giraffa* (Hemiptera: Reduviidae), a web invading, araneophagic assassin bug from Australia. *New Zealand Journal of Zoology,* 38, 297-316.
2. Soley, F. G., & Taylor, P. W. (2012). Araneophagic assassin bugs choose routes that minimize risk of detection by web-building spiders. *Animal Behaviour,* 84, 315-321.
3. Soley, F. G., & Taylor, P. W. (2013). Ploys and counterploys of assassin bugs and their dangerous spider prey. *Behaviour,* 150, 397-425.
4. Wirth, E., & Barth, F. G. (1992). Forces in the spider orb web. *Journal of Comparative Physiology A*., 171, 359-371.
